# A descriptive study of suspected perinatal asphyxia at Mitchells Plain District Hospital: A case series

**DOI:** 10.4102/safp.v62i1.5112

**Published:** 2020-10-09

**Authors:** Johannes P.J. Stofberg, Graeme W. Spittal, Tracey Hinkel, Tasleem Ras

**Affiliations:** 1 School of Public Health and Family Medicine, University of Cape Town, Cape Town, South Africa; 2 Department of Paediatrics, Mitchells Plain Hospital, Cape Town, South Africa; 3 Department of Obstetrics and Gynaecology, Mitchells Plain Hospital, Cape Town, South Africa

**Keywords:** hypoxic ischaemic encephalopathy, perinatal asphyxia, perinatal care, district healthcare, quality of care

## Abstract

**Background:**

South Africa aims to end all preventable deaths of children under the age of five as part of their commitment to the Sustainable Development Goals. More than half of these mortalities occur in the neonatal period with perinatal asphyxia as one of the leading causes. This study investigated and identified the characteristics of perinatal asphyxia and its contributing factors at a district hospital in Cape Town.

**Methods:**

A retrospective descriptive case series was performed and included all suspected cases of perinatal asphyxia referred from the Mitchells Plain District Hospital (MPH) to a specialised centre in the years 2016–2018. A data collection tool was used to extract information. Data were processed with Statistical Package for the Social Sciences 25 (SPSS) to produce descriptive statistics and to investigate associations between variables using the chi-square tests.

**Results:**

The study included 29 cases of suspected perinatal asphyxia. Ten cases (34.5%) had abnormal amplitude electroencephalograms (aEEGs) indicative of hypoxic ischaemic encephalopathy (HIE) and four (13.8%) demised before day seven of life. Non-operative deliveries (*p* = 0.005), lack of a doctor at the time of delivery (*p* = 0.004) and neonatal chest compressions (*p* = 0.044) were associated with abnormal aEEGs. Babies with Thompson score of equal to or more than 12 (*p* = 0.006), neonatal seizures (*p* = 0.036) and delayed arrival at referral hospital (*p* = 0.005) were associated with abnormal aEEG findings. Mortality was associated with Thompson score equal to or more than 12 (*p* = 0.007) and the need for neonatal intubation at delivery (*p* = 0.016).

**Conclusion:**

Significant reversible factors were identified in the peri- and post-partum periods. More capacitated staff would have the greatest impact on outcomes. The profile of HIE is exceedingly complex and challenges the resources and services of district level of care. Therefore, these factors should be targeted for future development and investment to improve outcomes from district hospitals.

## Introduction

Since 2005 there has been a decline in the global neonatal mortality rate from 36 per 1000 live births to 19 per 1000 in 2015. This marked the end of the Millennium Developmental Goals (MDG) initiative.^1^ These figures equate to an approximate decline from 4 million to 2.7 million annual neonatal deaths during this period.^1,2^ Despite these successes, neonatal mortality and morbidity prevails as one of the major global health concerns and the reduction of neonatal deaths has been disproportionally slower in comparison with deaths outside of the neonatal period.^3^

Goal 3 of the Sustainable Development Goals (SDG) aims to end all preventable deaths of children under the age of 5 years.^3^ Neonatal deaths constitute 45% in this group globally, and in South Africa (SA), it is estimated at 50%.^4,5^ According to the District Health Information System (DHIS), SA was close to the target neonatal mortality rate of 12 deaths per 1000 live births as set by the SDG, achieving 12.6 per 1000 live births in 2016.^6^ However, the South African Demographic Health Survey reports a higher neonatal mortality rate of 21 per 1000 live births.^7^ The difference between these two statistics is acknowledged by the authors. However, the reason for the discrepancy would require an in-depth analysis of their respective methodologies.

The three most prevalent causes of neonatal mortality in SA are complications of prematurity (47.9%), perinatal asphyxia (24.3%) and infections (11.6%).^6^ When one excludes the extremely low birth weight category (< 1000 g), perinatal asphyxia is the number one cause of death in neonates.^6^

Perinatal asphyxia is the term that describes a condition where there is decreased oxygenation of the foetus during the perinatal period (20 weeks gestation to day seven of life). When this period of decreased oxygenation is severe and prolonged, it may result in cerebral ischaemia severe enough to cause encephalopathy. This pathological process results in a syndrome, which is based on a combination of clinical and biochemical factors, referred to as hypoxic ischaemic encephalopathy (HIE).^8,9^

In SA, it is difficult to estimate the prevalence of perinatal asphyxia, and reports vary between 2.3 and 26.5 per 1000 live births.^10,11^ The wide range in prevalence can be explained by the complexity of this condition and numerous diagnostic criteria being utilised. In one study performed in 2011 at Chris Hani Baragwanath Hospital in Johannesburg, the incidence of perinatal asphyxia was found to range between 8.7 and 15.2 per 1000 live births. The same study suggested a mortality rate of 8.9% – 12.3% encompassing all severities of HIE.^12^ A study conducted in 2009 at Groote Schuur, Mowbray Maternity and New Somerset Hospitals showed a similar mortality rate of 13%.^13^

The diagnosis and management of neonates who suffered perinatal asphyxia with suspected HIE involves time-dependent intricate decision-making. In addition, specialised monitoring and treatment modalities are required, which can only be provided at specialised facilities. Timely referral depends on the attending clinician having a high index of suspicion of HIE prompting the use of validated scoring systems to assist with clinical decision-making.^14,15^ The gold standard diagnostic special investigation is an amplitude-electroencephalogram (aEEG). When conducted within the first 6 h of life, it has a sensitivity of 89% in predicting a normal outcome (no neurological sequelae) if the aEEG is found to be normal and a sensitivity of 94% in predicting a poor outcome with an abnormal aEEG.^16,17,18^ This modality is not available at district level of care and highlights the importance and urgency to refer patients who might have this condition.

The Saving Babies Report (2012–2013) estimates that nearly 50% of modifiable factors for perinatal asphyxia are associated with healthcare provider behaviour.^19^ These factors included failure to identify foetal distress (with and without foetal monitoring), delay in perinatal referral to a secondary or tertiary hospital, and inappropriate management of prolonged second stage of labour. Patient-related factors were identified as delay in seeking medical attention by the patient during labour, never initiating antenatal care, late antenatal booking and inappropriate action by the patient in reaction to warning signs. Over 50% of all perinatal asphyxia-related deaths occur at district level hospitals as opposed to community health centres (2.8%), regional (22.5%) or tertiary hospitals (23.9%).^6^ These figures suggest that there is potential of preventing a substantial number of perinatal asphyxia cases and HIE and therefore a significant amount of morbidity and mortality.

There is a lack of studies investigating the modifiable factors relating to perinatal asphyxia at district level hospitals. The aim of this study was to describe clinical care of the mother and baby dyad at Mitchells Plain District Hospital (MPH) to identify possible preventable contributors to perinatal asphyxia. This was achieved by the following objectives: firstly, by describing the antenatal course and peripartum care of the mothers who delivered babies with suspected perinatal asphyxia at MPH. Secondly, by describing the presence and extent of clinical features of perinatal asphyxia in neonates referred from MPH to Groote Schuur Hospital (GSH) or Mowbray Maternity Hospital (MMH) as suspected perinatal asphyxia. Finally, by describing the care of the mother-and-baby dyad in the perinatal period to identify possible modifiable factors, which could influence future practice and improve outcomes.

## Methodology

### Study design

This study was a retrospective descriptive case series with an analytical component.

### Setting

The primary research site was at MPH with extension to Groote Schuur (GSH) and Mowbray Maternity Hospitals (MMH). Mitchells Plain District Hospital is a large district-level hospital, which serves the greater Mitchells Plain community. It provides specialised obstetric services and is the referral centre for several midwife obstetric units (MOUs) in this area. Groote Schuur Hospital and MMH are the tertiary and secondary referral hospitals for MPH, respectively.

The Guidelines for Maternity Care in SA govern the patient profiles and their appropriate level of care across the referral platforms from primary to tertiary facility.^20^ These guidelines are accepted as the standard of care and are well established in this setting.

### Study population

The population included all the patients admitted to MPH where neonates were subsequently referred to GSH or MMH with suspected perinatal asphyxia during 2016–2018 (*n* = 29).

The definitive diagnosis of perinatal asphyxia or HIE requires an investigation (aEEG) and subsequent therapeutic hypothermia, which is only available at specialised facilities, and not at district level. Therefore, there exists a low threshold to refer any and all suspected cases, which meets the given criteria.

#### Inclusion criteria

Babies had one or more of the following factors present:

- A sentinel event in the perinatal course (abruptio placenta, cord prolapse, foetal bradycardia and prolonged second stage of labour)
- Apgar score of less or equal to seven at 5 min
- A need for prolonged resuscitation at birth (more than 10 min)
- Proven acidosis within the first hour of life, defined as a pH < 7 or a base deficit more than 10 mmol/L
- Clinical features of moderate to severe encephalopathy (abnormalities in activity, muscle tone, primitive reflexes, posture, seizures, autonomic system or level of consciousness)

Babies had all of the following factors:

- Be of 36 weeks gestation or more
- Have a birth weight of 1.8 kg or more
- Be referred from MPH to GSH or MMH

#### Exclusion criteria

Babies who met the above criteria and had one of the following criteria were excluded from the study:

- Have a significant and severe comorbid disease such as an unstable cardiac condition
- Severe congenital anomaly
- Required surgery within the first 3 days of life

### Data collection

A data-collection tool was developed by the authors of this article. It is based on existing literature (content validation) and reviewed by key role players at MPH and subsequently by specialist neonatologist and obstetricians (face validation). A pilot study was conducted with the data-collection tool prior to final adjustments. The pilot study included 10 cases (construct validation). No changes were made after the pilot study.

Case finding was performed by reviewing the folders on the electronic content management system (ECM) of all the babies transferred from the MPH-neonatal unit during 2016–2018. Twenty-nine cases met the inclusion criteria and were included in the study. The data-collection tool was used to extract data at MPH and the referral hospitals.

All the cardiotocograms (CTGs) were reviewed and described by specialist obstetricians. Interpretations by specialists were standardised by implementation of the National Institute for Health and Care Excellence (NICE) guidelines for CTG appraisal.^21^ At MPH, these guidelines are used in the clinical setting in review of CTGs and are visibly available on each CTG machine.

### Data analysis

Data were initially captured into Excel and subsequently transferred to Statistical Package for the Social Sciences 25 (SPSS) for analysis.^22^ Descriptive statistics were performed to obtain frequencies and proportions. Associations between categorical variables with outcome variables were determined by 2 × 2 tables and chi-square tests.

### Ethical consideration

Ethical approval was granted by the University of Cape Town Human Research Ethics Committee (Ref 644/2018). Formal permission was attained from the Western Cape Health Research Sub-directorate (WC_201905_022) as well as from MPH, GSH and MMH, respectively. This study adheres to the Declaration of Helsinki.^23^

## Results

A total of 33 cases that were referred from MPH with suspected perinatal asphyxia were identified between the years 2016 and 2018. Four cases were excluded from the study population because of either low birth weight or gestational age.

### Maternal demographics

Table 1 depicts the maternal demographics of the study population and the characteristics of their antenatal care. The median age of the mothers was 24 years, with more than half being primigravid. One mother was human immunodefeciency virus (HIV) positive and two were smokers. The median Body Mass Index (BMI) at the time of booking was 27.5 (20–44). Ninety per cent of mothers had a self-reported household income of < R100 000.00 ($6720.00) per annum and all mothers lived in urban areas.

**TABLE 1:** Maternal characteristics and early antenatal care.

Variable	*n*	%	Median	IQR
**Demographic**				
Mother age at presentation	-	-	24	21–29.5
Mother parity				
Nulliparous	16	55.2	-	-
Multiparous	13	44.8	-	-
Past obstetric complications				
Miscarriage	2	6.9	-	-
Termination of pregnancy	1	3.4	-	-
Preterm labour	1	3.4	-	-
Caesarean section	2	6.9	-	-
Mother BMI	-	-	27.5	22.5–31.8
Missing	4	13.8	-	-
18–24	7	24.1	-	-
25–29	9	31	-	-
30–39	7	24.1	-	-
> 40	2	6.9	-	-
Mother HIV status				
Negative	28	96.6	-	-
Positive	1	3.4	-	-
Smoker				
Smoker	2	6.9	-	-
Non-smoker	27	93.1	-	-
**Early antenatal care**				
Gestational age at booking	-	-	21	16–25.5
< 12	5	17.2	-	-
13–26	19	65.1	-	-
27–40†	5	17.2	-	-
Number of antenatal visits attended	-	-	5	3–6
< 5	12	41.4	-	-
≥ 5	17	58.6	-	-
Gestational age at presentation	-	-	39	37.5–39.5
36–40	24	82.8	-	-
> 41	5	17.2	-	-
First care facility				
Midwife obstetric unit	19	65.1	-	-
Mitchell’s Plain Hospital	10	34.5	-	-

IQR, interquartile range; BMI, Body Mass Index; HIV, human immunodeficiency virus.

† , Two mothers presented in this category who were unbooked.

### Early antenatal care

The median gestational age at booking was 21 weeks, with 17.2% mothers in this study booking before 12 weeks. Two mothers were unbooked and 58.6% had five visits or more. Nineteen mothers were initially presented to the MOU, and 10 mothers were presented to MPH directly. One baby was born at home and one at the MOU before presenting to MPH. Spontaneous labour occurred in 82.7% of mothers and 6.9% of mothers were induced.

### Peripartum care

Table 2 describes the peripartum course and care from the time of admission. A total of 10 (52.63%) of the 19 patients referred from the MOU were referred on the premise of possible foetal compromise. This decision was based on clinical features such as abnormal foetal heart rate on auscultation, meconium stained liquor or decreased foetal movements. Some of these mothers also had complications such as prolonged rupture of membranes or delayed second stage of labour along with foetal compromise. For the purpose of this study the suspicion of foetal compromise was given priority as reason for referral. Failure of progress in labour (15.8%) and delayed second stage (15.8%) were the second most common reasons for referral. The partogram was used in nine of the referred mothers; however, it was deemed to be inaccurate in 22.2% of cases when assessed by the receiving clinician. Intrapartum resuscitation was performed in most cases with 89.6% receiving intravenous fluid and 78.9% a urinary catheter. A single patient received tocolysis, as indicated by severe foetal distress. Left lateral position and oxygen administration were poorly documented, despite it being a standard practice. Priority-one transfer (highest priority) was booked for 42.2% of the transfers. The median transfer time was 72 min from the MOU to MPH.

**TABLE 2:** Course of clinical care at midwife obstetric units and Mitchells Plain District Hospital from admission.

Variable	*n*	%	Median	IQR
**Characteristics at MOU (*n* = 19)**				
Partogram used				
Yes	9	47.4	-	-
Used incorrectly	2	22.2	-	-
No	10	52.6	-	-
Primary reason for referral to MPH				
Possible foetal compromise	10	52.63	-	-
Failure to progress	3	15.8	-	-
Delayed second stage of labour	3	15.8	-	-
Breech in labour	1	5.3	-	-
Other	1	5.3	-	-
Prolonged rupture of membranes	1	5.3	-	-
Suspicion of foetal compromise				
Yes	10	52.36	-	-
No	9	47.37	-	-
Action taken				
IV fluid	17	89.5	-	-
Position (left lateral documented)	0	0	-	-
Urinary catheter	15	78.9	-	-
Ambulance priority level				
Not documented	5	26.3	-	-
Regular ambulance	1	5.3	-	-
Urgent ambulance	5	26.3	-	-
Flying squad	8	42.1	-	-
Time from referral to MPH arrival (min)	-	-	72	45–110
Characteristics at MPH				
Time from triage to first assessment by a doctor (min)	-	-	60	22.5–94
Doctor attending intrapartum care				
No	6	20.7	-	-
Yes	23	79.3	-	-
Foetal presentation				
Cephalic	26	89.7	-	-
Breech	3	10.3	-	-
Rupture of membranes (ROM)				
No rupture (C/S)	5	17.2	-	-
Spontaneous	19	65.1	-	-
Artificial	5	17.2	-	-
Duration of ROM (min)	-	-	360	120–765
Augmentation of labour with oxytocin				
Yes	5	17.2	-	-
No	24	82.8	-	-
Suspicion of foetal compromise				
Missing	1	3.4	-	-
Yes	25	86.2	-	-
No	3	10.3	-	-
Actions taken				
IV fluid	26	89.7	-	-
Position (left lateral documented)	9	31	-	-
Urinary catheter	21	72.4	-	-
Tocolysis	1	3.4	-	-
Doctor present at delivery				
No	10	34.5	-	-
Yes	19	65.1	-	-
Mode of delivery				
Caesarean	10	34.5	-	-
Vaginal	19	65.1	-	-
Complications of delivery				
None	8	27.6	-	-
Foetal distress	9	31	-	-
Delayed second stage of labour	5	17.2	-	-
Breech	3	10.3	-	-
Shoulder dystocia	3	10.3	-	-
Antepartum haemorrhage	1	3.4	-	-
Episiotomy performed	7	24.1	-	-
**Caesarean section details (*n* = 10)**				
Indication				
Foetal distress	6	60	-	-
Failure to progress	1	10	-	-
Failed assisted delivery	1	10	-	-
Antepartum haemorrhage	1	10	-	-
Elective	1	10	-	-
Time from decision to delivery (minutes)	-	-	45	34–66
Reason for delay†				
No delay	3	30	-	-
Not documented	2	20	-	-
Theatre not available	2	20	-	-
Surgeon not available	2	20	-	-
Urgency not recognised	1	10	-	-

IQR, interquartile range; IV, intravenous fluid; MOU, Midwife Obstetric Units; MPH, Mitchells Plain District Hospital; ROM, rupture of membranes.

† , Delay: more than 45 min.

At MPH, triage and midwife assessments occurred simultaneously in most cases. The longest delay was 50 min. Foetal presentation was cephalic in 90% of cases and the median duration of rupture of membranes (ROM) was 360 min. Sixty-five per cent had spontaneous ROM, 17% of membranes were ruptured artificially and the remainder were unruptured prior to C/S. Seventeen per cent received oxytocin augmentation of labour and a total of 65% delivered vaginally. There were three (10%) assisted deliveries performed with vacuum extraction. One was successful, and one failed, however delivered vaginally after bilateral episiotomy was performed. The last assisted delivery failed and was delivered via caesarean section (C/S).

The median time from decision of C/S to delivery was 45 min (IQR 23–97) and included one elective C/S. Occupied theatres and unavailability of the surgeon were the two main reasons for delay.

### Cardiotocogram characteristics

Table 3 demonstrates the particulars of intrapartum foetal monitoring with CTG. Monitoring with CTGs were performed in 86% of the cases and had a median time of 10 min from triage to first CTG. In 32% of the cases clinicians did not document any interpretation and in 28% they used nomenclature other than that of NICE guidelines. Comparing the interpretation of the CTGs by the attending clinician with specialist obstetrician’s interpretation applying NICE guidelines, only 32% of the attending clinicians were correct. According to obstetrician’s NICE guideline interpretation 68% of the CTGs were pathological.

**TABLE 3:** Cardiotocogram characteristics.

CTG details	*n*	%	Median	IQR
CTG used				
Yes	25	86.2	-	-
No	3	10.3	-	-
Missing	1	3.4	-	-
Triage to CTG (min)	-	-	10	3:40–16
**NICE CTG descriptions by attending clinicians (*n* = 25)**				
Normal or reassuring	7	28	-	-
Suspicious	1	4	-	-
Pathological	2	8	-	-
Description not according to NICE guidelines	7	28	-	-
None	8	32	-	-
CTG interpretation according to NICE†				
Correct	8	32	-	-
Incorrect	17	68	-	-
CTG NICE description†				
Normal/reassuring	4	16	-	-
Suspicious	2	8	-	-
Pathological	17	68	-	-
Inconclusive	2	8	-	-
Correct action taken on CTG				
Yes	14	56	-	-
No	11	44	-	-

CTG, cardiotocogram; IQR, interquartile range; NICE, National Institute for Health and Care Excellence.

† , As interpreted by specialist obstetrician.

Seventeen per cent of the deliveries occurred on weekdays between 07:30 and 16:30. During these times a full complement of staff is on duty. More than 80% of deliveries occurred during after-hours where there is only one medical officer and an intern on duty with one theatre available, which is shared with the surgical department.

**FIGURE 1:**
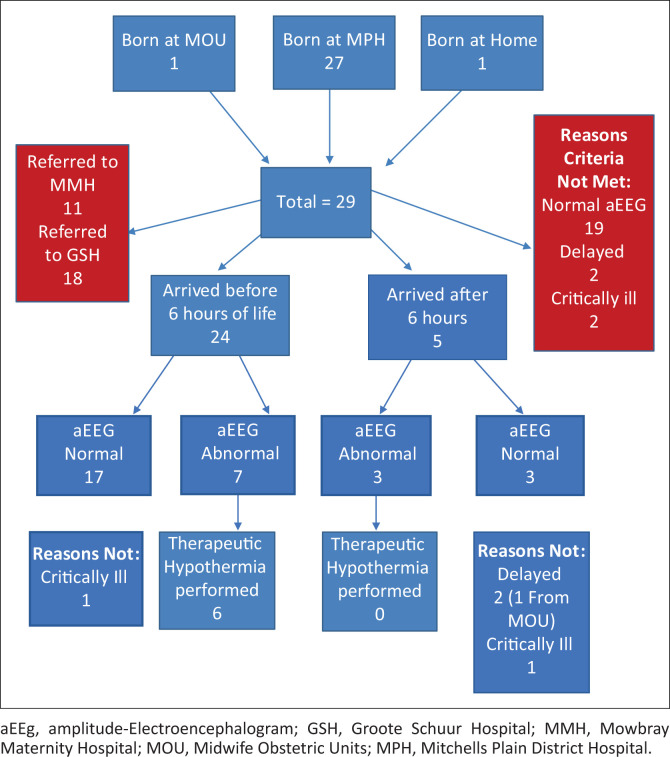
Overview of outcomes.

Figure 1 depicts an overview of the outcomes of the babies. Twenty-seven of the babies were born at MPH, one at home and one at the MOU. Twenty-four babies arrived at the referral hospital (GSH or MMH, respectively) before 6 h of life. Seven had abnormal aEEGs and met criteria for therapeutic hypothermia. One was excluded because of critical ill health.

Table 4 depicts the clinical details and course of the first 7 days of life of the babies. Fifty-five per cent of the babies were male and the median gestational age and weight were 39 weeks and 3 kg, respectively. The median 5-min Apgar scores were six, pH 7.1 and base excess –13.9. One baby received adrenaline during resuscitation; however, 10 babies required chest compressions and 11 babies were intubated. Ten babies had documented seizures that were all treated with phenobarbitone. The median HIE score was nine.

**TABLE 4:** Details describing the babies’ clinical and therapeutic course from birth to day seven of life.

Features	*n*	%	Median	IQR
Female sex	13	45	-	-
Gestation (weeks)	-	-	39.0	37.5–39.5
Weight (kilograms)	-	-	3.0	2.7–3.6
Presence of documented foetal compromise	11	38	-	-
**Apgar scores**				
1 min	-	-	3.0	1.0–4.0
5 min	-	-	6.0	5.0–7.0
10 min	-	-	8.0	6.0–9.0
**Blood gas in the first hour of life**				
pH	-	-	7.1	7.0–7.2
Lactate (mmol/L)	-	-	11.0	7.5–15.0
Base excess	-	-	−13.9	−18.4–10.9
**Management**				
Intubation performed	11	37.9	-	-
Adrenaline administered	1	3.4	-	-
CPAP required	13	44.8	-	-
Missing	1	3.4	-	-
Total time CPAP required (h)	-	-	4	2.6–6.0
IPPV required (min)	24	82.8	9	5–14
Chest compressions required (min)	10	34.5	2.0	0.9–2.8
Time to spontaneous respiration (min)	-	-	5.0	2.5–8.5
Missing	8	27.6	-	-
Time to heart rate > 100 beats (min)	-	-	45 s	0.01–5.00
Missing	1	3.4	-	-
**Post-resuscitation management**				
Intravenous fluids (potassium free)	29	100	-	-
Intravenous antibiotics	29	100	-	-
Inotropes	4	13.8	-	-
Thompson HIE score	-	-	9.0	6.5–11.5
Presence of seizures†	10	34.5	-	-
Time from birth till arrival at referral centre (h)	-	-	4.8	4–5.75
aEEG before 6 h of life	24	82.8	-	-
aEEG after 6 h of life	5	17.2	-	-
Delayed presentation	1	3.4	-	-
EMS delay	2	6.9	-	-
Delay in discussion with referral site	2	6.9	-	-
aEEG findings	29	100	-	-
Continuous normal voltage	19	65.1	-	-
Discontinuous normal voltage	4	13.8	-	-
Grossly abnormal	3	10.3	-	-
Burst suppression	1	3.4	-	-
Status epilepticus	1	3.4	-	-
Continuous low voltage	1	3.4	-	-
Alive day 7	25	86.2	-	-

aEEG, amplitude electroencephaologram; APGAR, Appearance, Pulse, Grimace, Acticity, Respiration; CPAP, continuous positive airway pressure; EMS, Emergency Medical Services; HIE, hypoxic ischaemic encephalopathy; IPPV, intermittent positive pressure ventilation; IQR, interquartile range; pH, power of hydrogen.

† , All seizures were treated with phenobarbitone, according to standard of care.

Transfer from MPH to the referral hospitals took on average 4.8 h (median) and directly resulted in two babies arriving after 6 h of life. All babies received aEEG monitoring despite five arriving after the therapeutic window. The aEEGs were shown to be normal in 19 babies and abnormal in 10 babies. Four babies with abnormal aEEGs did not met the criteria for therapeutic hypothermia as two babies were delayed and two babies were critically ill. On day seven of life, 92% of babies had good clinical outcomes. There were four deaths in total (13.8%).

Table 5 represents the statistical analysis of the data collected. It depicts associations between variables and abnormal aEEG results and mortality, respectively. Significant associations were shown between normal aEEG and vaginal deliveries (*p* = 0.005), the presence of a doctor at time of delivery (*p* = 0.004) and clinical features, including an HIE score of more than 12 (*p* = 0.006) and seizures (*p* = 0.036). Neonatal arrival at the referral hospital within 6 h was associated with normal aEEGs (*p* = 0.005).

**TABLE 5a:** Associations between variables and outcomes (abnormal amplitude electroencephalograms and mortality) represented by cross-tabulations and chi-square tests.

Variable	Abnormal aEEG (*n* = 10)		Normal aEEG (*n* = 19)		Total (*n* = 29)	*p*
	*n*	%	*n*	%		
**Cross-tabulations (chi-squared tests) observed and expected counts**						
BMI category						0.264
BMI > 30	2	3.2	7	5.8	9	-
BMI ≤ 30	7	5.8	9	10	16	-
MSL present						0.840
Yes	4	5.2	11	9.8	15	-
No	6	4.8	8	9.2	14	-
Early antenatal booking (1st trimester)						0.775
Yes	2	1.7	3	3.3	5	-
No	8	8.3	16	15.7	24	-
Antenatal visits five or more						0.14
Yes	4	5.9	13	11.1	17	-
No	6	4.1	6	7.9	12	-
Nulliparous						0.144
Yes	6	5.5	10	10.5	16	-
No	4	4.5	9	8.5	13	-
Partogram					19	0.130
Yes	5	3.3	4	5.7	9	-
No	2	3.7	8	6.3	10	-
Suspected foetal distress at MOU					19	0.663
Yes	4	4.1	7	6.9	11	-
No	3	2.9	5	5.1	8	-
Suspicious or pathological CTG						0.699
Yes	7	6.7	13	13.3		-
No	1	1.3	3	2.7		-
CTG: Correct interpretation					25	0.265
Yes	2	2.6	6	5.4	8	-
No	6	5.4	11	11.6	17	-
Doctor was present at delivery						0.004
Yes	3	6.6	16	12.4	19	-
No	7	3.4	3	6.6	10	-
Mode of delivery						0.005
Vaginal	10	6.6	9	12.4	19	-
Caesarean	0	3.4	10	6.6	10	-
Delayed second stage of labour						0.947
Yes	2	2.1	4	3.9	6	-
No	8	7.9	15	15.1	23	-
After hours delivery						0.424
Yes	9	8.3	15	15.7	24	-
No	1	1.7	4	3.3	5	-
Chest compressions						0.044
Yes	1	3.4	9	6.6	10	-
No	9	6.6	10	12.4	19	-
Baby’s pH ≤ 7.15 in first hour of life					28	0.907
Yes	5	5.1	11	10.9	16	-
No	4	3.9	8	8.1	12	-
Baby’s lactate ≥ 11					28	0.686
Yes	4	4.5	10	9.5	14	-
No	5	4.5	9	9.5	14	-
HIE score ≥ 12					28	0.006
Yes	5	2.1	1	3.9	6	-
No	5	7.9	17	14.1	22	-
Clinical seizures						0.036
Yes	6	3.4	4	6.6	10	-
No	4	6.6	15	12.4	19	-
NICU arrival before 6 h of life						0.005
Yes	**4**	7.2	17	13.8	21	-
No	**6**	2.8	2	5.2	8	-
Baby alive on day 7						0.066
Yes	7	8.6	18	16.4	25	-
No	3	1.4	1	2.6	4	-

aEEG, abnormal amplitude electroencephalograms; BMI, body mass index; CTG, cardiotocograms; HIE, hypoxic ischaemic encephalopathy; MOU, Midwife Obstetric Units; MSL, meconium stained liquor; NICU, Neonatal Intensive Care Unit.

† , One patient’s hypoxic ischaemic encephalopathy score was not documented.

‡ , Only including pathological or suspicious cardiotocograms.

**TABLE 5b:** Associations between variables and outcomes (abnormal amplitude electroencephalograms and mortality) represented by cross-tabulations and chi-square tests.

Variable	Alive day 7 *n* = 24		Demised day 7 *n* = 4		Total *n* = 28	*p*
	*n*	%	*n*	%		
**Cross-tabulations (chi-squared tests) observed and expected counts**						
Early antenatal booking (1st trimester)						0.568
Yes	4	4.3	1	0.7	5	-
No	20	19.7	3	3.3	23	-
Antenatal visits 5 or more						0.417
Yes	13	13.7	3	2.3	16	-
No	11	10.7	1	1.7	12	-
Delayed second stage of labour						0.687
Yes	4	4.1	1	0.7	5	-
No	20	19.7	3	3.3	23	-
Baby’s pH ≤ 7.15 in the first hour of life					28	0.687
Yes	13	13.7	3	2.3	16	-
No	11	10.3	1	1.7	12	-
Baby’s lactate ≥ 11					28	0.098
Yes	10	12	4	2	14	-
No	14	12	0	2	14	-
HIE score ≥ 12†					27	0.007
Yes	3	5.3	3	0.7	6	-
No	21	18.7	0	2.3	21	-
Intubation of baby					28	0.016
Yes	7	9.4	4	1.6	11	-
No	17	14.6	0	2.4	17	-
NICU arrival before 6 h of life						0.052
Yes	20	18.1	1	2.9	21	-
No	5	6.9	3	1.1	8	-
Suspicious or pathological CTG‡					24	0.546
Yes	15	15.8	3	2.3	18	-
No	6	5.3	0	0.8	6	-
Chest compressions						0.265
Yes	10	8.6	0	1.4	10	-
No	14	15.4	4	2.6	18	-

aEEG, abnormal amplitude electroencephalograms; BMI, body mass index; CTG, cardiotocograms; HIE, hypoxic ischaemic encephalopathy; MOU, Midwife Obstetric Units; MSL, meconium stained liquor; NICU, Neonatal Intensive Care Unit.

† , One patient’s hypoxic ischaemic encephalopathy score was not documented.

‡ , Only including pathological or suspicious cardiotocograms.

Mortality was shown to have correlation with the need for neonatal intubation (*p* = 0.16) and a HIE score of more than 12 (*p* = 0.007).

## Discussion

This study aimed to describe the characteristics of the antenatal and perinatal care of the mother-baby dyad and the clinical features of HIE in the babies referred with suspected perinatal asphyxia. The purpose is to identify the contributing factors, which could be preventable.

### Antenatal care

Current guidelines dictate booking prior to 12 weeks gestation and for uncomplicated antenatal course (low-risk cases), a minimum of five antenatal visits.^20^ Sixty-six per cent of mothers obtained the recommended five or more antenatal visits. Seventeen per cent of mothers booked in the first trimester, and the median gestation at booking was 21 weeks. Two mothers were unbooked. Despite late booking gestation, mothers had frequent visits that accounts for the majority of patient’s having adequate antenatal attendance. A study from Nigeria found mothers who had no antenatal care to have three times the risk of HIE compared with mothers with adequate care.^24^ Contrarily, antenatal attendance and gestation at booking showed no association with abnormal aEEG or mortality in this study. Nulliparity and raised BMI have been linked with increased risk of HIE.^25,26,27,28^ Fifty-five per cent of mothers were nulliparous, although they did not have significant association with abnormal aEEG (*p* = 0.144) nor mortality (*p* = 0.299), as can be seen in Table 5. The median booking BMI of mothers was 27.5 and included two patients with BMIs above 40. However, BMI had no significant association with outcomes. In addition, smoking, maternal age and previous obstetric complications were not found to be significant.

There was only one mother included in the study who was HIV positive, despite the estimation of up to 20% of babies delivered at MPH being HIV exposed. She was virally suppressed and had good antenatal attendance. It is possible that the lower than expected incidence of mothers with HIV in the study population could be explained by the promotion of antenatal care by specialised HIV clinics, subsequently resulting in earlier diagnosis of pregnancy, initiation of antenatal care and improved antenatal care attendance. A study performed in Lesotho supports this correlation with known HIV-positive women and antenatal care.^29^

### Perinatal care

The antenatal service provided in SA is based on a robust referral system. There are clear guidelines on the appropriate level of care for the spectrum of clinical complexity. Levels of care involve an MOU, district hospital (MPH), secondary (MMH) and tertiary hospitals (GSH). Guidelines suggest all low-risk pregnancies to be managed entirely at MOU level of care.^20^ This explains why the majority (65.5%) of the study population initially presented to the MOU. Furthermore, current guidelines dictate highest priority emergency transport whenever foetal compromise is suspected. However, this level of transport was booked for only 42.1%. Not all mothers with suspected foetal distress (52.36%) were transferred via the fastest means available. Although the MOU is within 5 km of MPH, the median transfer times were 72 min. Intrapartum resuscitation was performed appropriately in nearly all the patients.

Mitchells Plain District Hospital staff related factors were important. A doctor present at delivery was shown to be a protective factor (aEEG *p* = 0.004). Midwife assessments of patients were rapid. However, time to doctor assessments was significantly longer (median 60 min). Six of the mothers (20.7%) were not assessed by a doctor at any time during admission. More than 80% of deliveries occurred after-hours; however, the time of delivery showed no statistical significance (aEEG *p* = 0.424, mortality *p* = 0.55.) as an independent variable. Similarly, a study conducted in Sweden indicated no associated risk with perinatal asphyxia and time of birth.^30^ This study was conducted in a developed country and at a tertiary hospital so direct comparisons are unlikely transferrable to our study population.

Caesarean section was performed in 10 cases (34.5%) and had statistically significant (*p* = 0.005) better outcome than the babies born via normal vertex deliveries (NVD). None of these babies had abnormal aEEGs, suggesting that timeous operative delivery in this population was a protective factor. There were three assisted deliveries, of which one led to an outcome of an abnormal aEEG. Studies have shown strong associations with obstetric emergencies (cord prolapse, ruptured uterus, shoulder dystocia, abruptio placenta) and perinatal asphyxia.^25,27,31^ Similarly, our patient population included emergencies. However, because of infrequent occurrence statistical associations could not be analysed. In contrast to other studies,^27,31^ delayed second stage of labour in our population were not found to be significant (aEEG *p* = 0.947, death *p* = 0.687).

Cardiotocograms monitoring is the mainstay in identifying foetal compromise. Correlation between pathological CTGs and clinical outcomes were not shown to be statistically significant in this study. However, concerns remain that attending clinicians were able to identify the foetal heart rate patterns correctly in only 32% of the cases. Correct identification of pathological patterns (62.1%) could have prompted expediting delivery via C/S and potentially altered outcomes.

### Neonatal factors relating to perinatal asphyxia

Chest compressions appeared to be a protective factor (aEEG *p* = 0.044). This speaks about adequate resuscitation of babies who received chest compressions. Therefore, the lack of chest compressions in other babies when indicated, could implicate an association with perinatal asphyxia. The same was not true for the need to intubate during resuscitation. Intubation was associated with higher mortality (*p* = 0.016). This correlation with intubation and mortality could suggest that these babies were saved from a terminal clinical condition and that their prognosis remained unchanged despite being referred. On the contrary, this correlation could infer resuscitation was inadequate and therefore necessitated intubation.

Our study is consistent with the findings from other studies, which have shown that the Thompsons HIE scoring system is useful for predicting outcome.^13,15^ An HIE score of more than 12 was associated with abnormal aEEG (0.006) and supports the Thompson score as a relevant tool in our setting. In addition, mortality (13.7%) of this study population was similar to that of other local populations and was associated with a Thompson score of more than 12 (*p* = 0.007).^12,13^ These findings add to the validity of the study, displaying correlation with a well-established screening tool and comparability with similar studies.^13,15^ Finally, clinical seizures at the referral site were predictive of abnormal aEEGs (*p* = 0.036). This correlation speaks about the consistency of accurately identifying an independent, clinical variable at district level of care, which can be used to predict HIE.

Delay in arrival at the referral centre was another predictive factor for abnormal aEEGs (*p* = 0.005), however not for mortality (*p* = 0.052). The reasons for delay were delayed presentation, delayed diagnosis and delay with transfer (EMS). Emergency Medical Services is faced with many challenges, including limited vehicle fleet and personnel, dangerous areas classified as red zones and an extremely high workload. This could have played a role. However, it is definitely not the only factor at play. The management of babies with suspected perinatal asphyxia is complex with many steps where care can be altered. It is vital to have a multifaceted approach to management and any change in management needs to be adopted throughout the health system.

Identification of perinatal asphyxia is vital to allow opportune referral and treatment. Identification relies on the attending clinician’s interpretation of clinical and biochemical features of the newborn baby. First, Apgar scores are often used as an indicator of concern.^32^ Conversely, our study did not find significant associations with 5-min Apgars < 5 (aEEG *p* = 0.291, death *p* = 0.107). Second, a blood gas analysis within the first hour of life can be used to guide diagnosis.^32^ In particular, the pH and lactate values are used. However, these variables had no relation with outcomes in this study.

## Limitations

Although the study was inclusive of all cases over the span of 3 years, the study population is small and representative of a singular study site. The study did not include mild forms of asphyxia, which did not require referral, nor did it include unrecognised cases from the MOU. Furthermore, fresh stillborn babies and neonatal deaths at MPH that might have been as a result of asphyxia were not included in this study. Although diligence was taken to promote scientific validity, the strength of the study relied upon adequate recording of data in clinical notes. These factors may have influenced the results of this study.

## Recommendations

The authors of this study would propose the following:

- The findings highlight the importance of early identification of high-risk labour and the appropriate management thereof. This may be achieved by
  ■ ongoing training of all front-line obstetric staff on CTG interpretation and management.
  ■ heightened foetal surveillance with continuous CTG monitoring, optimised intrapartum resuscitation, removal of precipitating factors and early consideration for operative delivery in high risk cases with suspected foetal distress.
- Training in neonatal resuscitation – across the care team
- Recognition and management of neonatal seizures were managed well. As this is a significant identifier of HIE, ongoing training in the use of the Thompson score and the recognition and management of neonatal seizures, should be emphasised.
- Use tools to standardise care within the referral pathway and to ensure appropriate booking of priority ambulances and enable the safe and timeous transport of the mother-baby dyad. Tools may include the physical referral form which is completed for every referral at MPH from the MOU. This will identify possible high-risk patients who could either be referred to a higher level of care, indicate the priority of transfer required and allow for preparation in anticipation of a particular patient scenario. Other tools may access to the EMS booking website where the clinician may book transport himself or herself.
- Further research is required:
  ■ At other district level facilities to support and further explore the findings of this study. As well as, to broaden the study population.
  ■ Throughout the referral pathway (MOU to tertiary hospital) investigating staff experiences and working conditions.
  ■ Investigating the impact and need for doctors present at the time of high-risk deliveries.

## Conclusion

This study highlights the complex challenges in care pathways in facilitating the timely diagnosis and management of perinatal asphyxia, particularly at district level. It has shown the necessity for having a low threshold for referral demanded by the high morbidity and mortality associated with perinatal asphyxia. Furthermore, the diagnosis and management of perinatal asphyxia are complicated by the deficiency of resources at this level of care. Although antenatal factors are important, significant reversible factors were identified in the peri- and post-partum periods. Adequate number of staff with the competency to identify foetal compromise, manage obstetric emergencies and neonatal resuscitation would have the greatest impact on outcomes. The highest burden of HIE lies at district level of care and future investment should target these factors to improve outcomes.
